# Spatial–Spectral Analysis of Hyperspectral Images Reveals Early Detection of Downy Mildew on Grapevine Leaves

**DOI:** 10.3390/ijms231710012

**Published:** 2022-09-02

**Authors:** Virginie Lacotte, Sergio Peignier, Marc Raynal, Isabelle Demeaux, François Delmotte, Pedro da Silva

**Affiliations:** 1 Univ Lyon, INSA Lyon, INRAE, BF2I, UMR203, F-69621 Villeurbanne, France; 2 IFV, UMT Seven, F-33140 Villenave d’Ornon, France; 3 SAVE, INRAE, Bordeaux Sciences Agro, Univ. Bordeaux, F-33140 Villenave d’Ornon, France

**Keywords:** bioinformatics, hyperspectral imaging, *Plasmopara viticola*, grapevine, disease management, early detection, Support Vector Machine, classification, automation

## Abstract

Downy mildew is a highly destructive disease of grapevine. Currently, monitoring for its symptoms is time-consuming and requires specialist staff. Therefore, an automated non-destructive method to detect the pathogen before the visible symptoms appear would be beneficial for early targeted treatments. The aim of this study was to detect the disease early in a controlled environment, and to monitor the disease severity evolution in time and space. We used a hyperspectral image database following the development from 0 to 9 days post inoculation (dpi) of three strains of *Plasmopara viticola* inoculated on grapevine leaves and developed an automatic detection tool based on a Support Vector Machine (SVM) classifier. The SVM obtained promising validation average accuracy scores of 0.96, a test accuracy score of 0.99, and it did not output false positives on the control leaves and detected downy mildew at 2 dpi, 2 days before the clear onset of visual symptoms at 4 dpi. Moreover, the disease area detected over time was higher than that when visually assessed, providing a better evaluation of disease severity. To our knowledge, this is the first study using hyperspectral imaging to automatically detect and show the spatial distribution of downy mildew on grapevine leaves early over time.

## 1. Introduction

Downy mildew, caused by the obligate oomycete *Plasmopara viticola*, is a highly destructive disease of grapevine *Vitis vinifera* L. [1,2]. The infection cycle of *P. viticola* begins during the spring with the germination of oospores (resting stage of the pathogen resulting from sexual reproduction) with the help of the warm and humid weather [1,2]. The sporangia disperse onto the vegetation by rainfall and release zoospores. The gem tube of zoospores enters the plant through the leaf stomata, grows into the plant tissue and produce new sporangia [1,2]. The infection is characterized by oil spots on the adaxial leaf surface and whitish downy covers on the abaxial surface, which are tufts of sporangia [1,2,3,4]. With favourable conditions, downy mildew can quickly infect the entire crop. Early infection of bunches can lead to significant crop loss (complete destruction of yield), whereas leaf infection affects the source–sink relationship in the vine. The control of downy mildew is therefore a major issue for winegrowers in regions where there is spring rainfall, especially in light of simulations predicting a potential increase in disease incidence due to climate change in the next decades [4].

Since the emergence of the disease, the control of downy mildew has been achieved with multiple fungicide applications, including the Bordeaux mixture based on copper discovered in 1885 by PMA Millardet [5]. Currently, the difficulty in curing the disease leads winegrowers to spray fungicides at any time there are favourable conditions to prevent the disease [6]. This solution is not reliable as it is costly and can endanger human health, pollute the environment, and develop fungi resistance. Thus, several institutions promote the reduction in agrochemicals, such as the French government, with the Ecophyto II+ plan supported by Europe that aims to reduce 50% of the use of agrochemicals by 2025 [7]. To achieve this goal, it is possible to combine different solutions, for example with the choice of more resistant varieties, the use of natural fungicides [3,8], or early detection of the disease. Previously, to detect the disease in the field, winegrowers would follow a sampling procedure from different strategic points and make a visual plant disease assessment. The plant disease severity can be expressed on a scale corresponding to a percentage of the area of the sampling unit, for example, a leaf displaying symptoms [9]. However, visual assessment can be time-consuming and subject to rater error because of variable perception of light and color or hard identification of the border between the infected and non-infected areas. The objective is to detect the symptoms as soon as possible considering the plant has already been affected and will soon be ready to infect other plants. Unfortunately, it has been established that a low disease severity remains invisible to the human eye [9].

Thereby, several optical sensors are being tested under laboratory conditions, followed by testing in the field, to make a continuous non-destructive monitoring of vineyards and to detect the presence of the pathogen on the crop before the onset of visible symptoms. These tools could help growers reduce their use of chemicals by helping them spray their crops at the right time and in the right place. Thermal sensors can assess the canopy temperature, which is correlated with plant stress. Along with diseases, other external factors such as water or heat stress can also influence the canopy temperature [10,11,12,13,14,15]. These confounding effects make thermal detection more difficult. Another method to detect diseases in plants is fluorescence imaging. The plant fluorescence varies according to photosynthetic activity and defence reactions and is measured by ultraviolet excitation on dark-adapted leaves [12,15,16,17,18]. This method is particularly efficient for a pre-symptomatic detection of fungal infections, especially on the abaxial side of the leaf for downy mildew in grapevine [6,19,20,21]. However, fluorescence imaging requires dark adaptation for the plants, which can compromise its use at a large scale [10,11,15]. Thermography and fluorescence imaging can detect early plant stress responses but cannot identify specific diseases, which is instead possible using RGB (Red-Green-Blue) wave bands, multi-, and hyperspectral sensors.

The RGB method consists of analysing the colour distribution and also the grey levels and texture of each pixel in a digital image [9,11]. Thus, it is possible to detect the presence and severity of pests [22,23] and diseases such as grapevine downy mildew in plants [24]. Nevertheless, this technique has its limits since the colours and shapes can get mixed up and its efficiency strongly depends on the quality and distance of the device. A solution is to use multi- and hyperspectral sensors. These sensors can measure the light reflectance spectrum of objects in the visible (400–700 nm), Near Infrared (NIR, 700–1000 nm), and Short-Wave Infrared (SWIR, 1000–2500 nm) range. Multispectral sensors usually focus on several relatively broad wave bands (RGB, NIR) whereas hyperspectral systems cover a broader range of wavelengths, providing a higher spectral resolution [11,17,25]. They can be imaging or non-imaging systems. Non-imaging systems are commonly used to calculate vegetation and disease indices from algorithms based on the reflectance of specific wavelengths. The Normalized Difference Vegetation Index (NDVI), reporting photosynthetic activity, is an indirect disease indicator since symptoms such as leaf yellowness and whitish cover have a well-known impact on photosynthesis [11]. Disease symptoms and their severity can be detected such as powdery and downy mildew on grapevine leaves [8,26,27]. Thus, spectral sensors can identify diseases and their symptoms by calculating the average reflectance over an area, but this method can mix multiple objects over the given area and compromise the results. The combination of spatial and spectral dimensions is therefore the best detection solution giving precise identification and location of the disease at a low or high severity. This is possible with Hyperspectral Imaging (HSI) [8,12,25,28,29,30,31].

HSI cameras are the latest innovative optical sensor to detect pests and diseases on crops [12,17,25]. HSI is still under research and only a few studies have been conducted on its efficiency in plant disease detection, but it has already proven its great potential [9,25]. HSI huge datasets usually require statistical analysis such as linear regression, Principal Component Analysis (PCA), Spectral Angle Mapper (SAM), and Support Vector Machine (SVM) classifications to extract the relevant information and obtain a high accuracy of disease detection. Thus, these statistical methods have been used to detect grey mould, early blight, and late blight on eggplant and tomato leaves [30,32,33,34,35]. In addition, some studies on sugar beet and wheat diseases focused on phenotyping of several diseases of a crop [29,36] or early detection of the disease by analysing its development in time and space [31,37], but the classification accuracy still needs to be improved. Finally, HSI has been used to phenotype downy mildew on several susceptible and resistant grapevine genotypes [8] and to detect by spatial–spectral analysis different infection levels of powdery mildew on wine bunches to prevent the infection of the coming harvest [38,39].

The aim of this study was (I) to explore the possibility in a controlled environment of identifying downy mildew on grapevine leaves, (II) to detect the disease before the first symptoms visually appear, and (III) to monitor disease severity evolution in time and space. To meet this goal, we used an HSI database following the daily development from 0 to 9 days post inoculation (dpi) of three strains of *P. viticola* inoculated on the leaves of a little susceptible grapevine cultivar, ‘Cabernet-Sauvignon’ [3]. Then, we extracted spatial–spectral data from symptomatic patches of hyperspectral images and developed an automatic detection tool based on Support Vector Machine (SVM) classifiers.

## 2. Materials and Methods

### 2.1. Plant Material and Pathogen Inoculation

The hyperspectral images used in this work came from a database publicly available at [40]. According to the information provided in the database, the leaves of *V. vinifera* L. cv ‘Cabernet Sauvignon’ came from cuttings grown in a glasshouse (SAVE laboratory, France). The cultivar appears to be mildly susceptible to *P. viticola* [3], which is fine for developing a tool capable of detecting disease in the most difficult settings, even when the plant shows mild symptoms. Three strains of *P. viticola* with different levels of virulence, INRA-Pv13, INRA-Pv42, and INRA-Pv45 were inoculated on leaves of ‘Cabernet Sauvignon’ while other leaves were kept healthy as a control. The inoculation was performed by placing three drops containing a sporangia suspension on the abaxial leaf surface. The disease incubation and development lasted 9 days at 22 °C in a growth chamber with a 12h photoperiod.

### 2.2. Visual Assessment of Downy Mildew on Grapevine Leaves

An expert inspection was carried out on the abaxial leaf surface to visually assess the disease symptom of whitish downy cover (sporangia) as a percentage of leaf area infected (Table 1). Disease severity scales based on a percentage of leaf area infected are widely used to assess plant disease severity in a quantitative way instead of descriptive scales which can be too subjective [9]. Early interval scales developed by Horsfall and Heuberger in 1942 [41] have been used due to their simple and suitable use for early symptom assessment.

### 2.3. Hyperspectral Imaging

**Figure 1:**
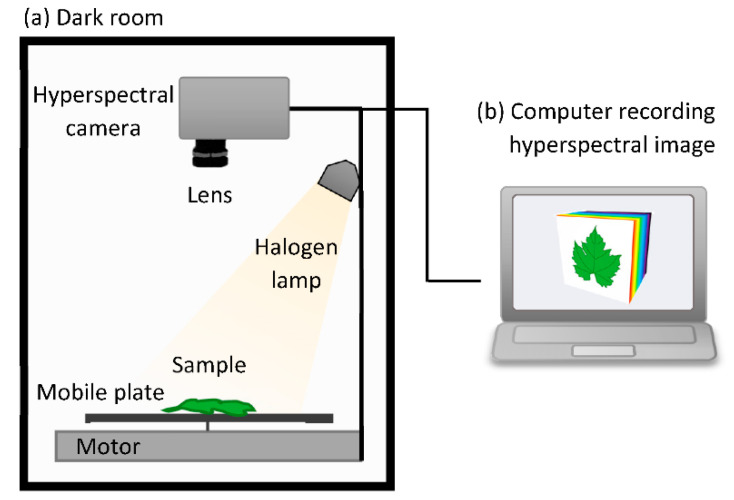
Schematic representation of the pushbroom hyperspectral imaging system used for the acquisition of the hyperspectral image database. (**a**) In a dark room with a halogen lamp as unique source of light, hyperspectral camera recorded the hyperspectral signature of each pixel of the sample moving under the lens (**b**) The system was controlled on a computer where all images were stored for later analysis. At the end of the recording, this resulted in a complete hyperspectral image, also called a hypercube, formed by the X and Y spatial axes and the Z spectral.

The hyperspectral images of infected leaves available in the database were taken daily, for each strain, from 1 to 9 dpi for 3 replicates, from 1 to 4 dpi for 2 replicates, and from 2 to 4 dpi for 5 replicates. Hyperspectral images of the healthy leaves used as control were also captured for 4 days with 5 replicates to show the absence of disease development in the latter, providing over 160 images. The images were acquired using an HSI camera Specim FX10 coupled with halogen lighting mounted on a Specim LabScanner (Specim, Spectral Imaging Ltd., Oulu, Finland), which was controlled using Specim’s LUMO Scanner Software Suite (2018 version, Specim, Spectral Imaging Ltd., Oulu, Finland) on a computer connected to the system (Figure 1). The camera had a spectral range of 400–1000 nm, corresponding to the visible and NIR ranges (VNIR), and a mean spectral resolution of 5.5 nm (224 bands). The system could record a hyperspectral image with a camera line of 1024 spatial pixels and spectral data acquisition along this line with a maximum frame rate of 327 frames per second (fps) full-frame. To ensure a controlled environment during the hyperspectral measurements, the HSI system was in a black room. Each hyperspectral image came with a dark and white reference for reflectance calibration. These two extra images were taken by closing an internal camera shutter and on a white calibration bar with a standard reflectance of 99%, respectively.

### 2.4. Hyperspectral Images Training Set Processing

**Figure 2:**
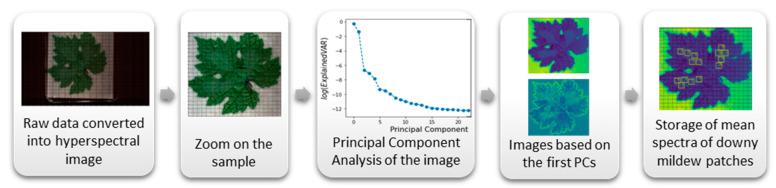
Hyperspectral image processing resulting in a downy mildew patches spectral library. The Principal Component Analysis (PCA) on the zoomed image pixels spectra, computed the percentage of variance carried by each Principal Component (PC). Then, the visualization of the zoomed image projected into the most important PCs subspaces revealed downy mildew on the leaves.

The hyperspectral image training set was prepared using a Jupyter Notebook script [42] relying on the Pandas [43], Numpy [44], Seaborn [45], Matplotlib [46], Spectral [47], and Scikit-learn [48] Python libraries. For each hyperspectral image, first, we converted the raw data into a hyperspectral reflectance image and zoomed in on the sample (Figure 2). Then, we applied a Principal Component Analysis (PCA) [49] on the zoomed image pixels spectra, computed the percentage of variance carried by each Principal Component (PC), and visualized the zoomed image projected into the most important PCs subspaces, revealing downy mildew on the leaves. The whitish downy cover gradually spread on the leaf and mixed with leaf spectral signature, and so it was not possible to apply a clustering algorithm on the pixels to capture a single cluster gathering the downy area. Thus, we selected multiple squared patches from representative parts of the leaves, avoiding overlaps between patches as much as possible, and stored their spatial–spectral data in a training library. Regarding the mildew-infected patches, expert inspection was carried out to select the patches of areas that we were highly sure was downy mildew, according to a previous visual assessment, corresponding to the disease severity scales 2 and 3 observed on the infected leaves between 4 and 9 dpi. Healthy leaf patches were selected randomly covering different parts of the leaves. Depending on the leaves and the quality of the images, different numbers of patches could be extracted per leaf. In total, the curated dataset contained 1386 patches from 62 leaves, among which 771 corresponded to patches from healthy leaves, and 615 to patches from parts of leaves severely infected by downy mildew. Finally, the reflectance of the images was normalized between 0, for no reflectance, and 1, for total reflectance, using the shutter and the white calibration images.

### 2.5. SVM-Based Downy Mildew Detection

To detect downy mildew in hyperspectral images, we used an SVM classifier with a Radial Basis Function (RBF) kernel. This binary classifier takes as an input the mean spectrum of a squared patch of *s* pixels width and aims at predicting the class membership of this patch, i.e., downy mildew-infected area or healthy area.

More formally, let $x_{i}\in X\;$ be a data point denoting the average spectrum of the patch *i*, and $X={\mathbb{R}}^{d}$ where d is the number of wave lengths in the dataset, and let $y_{i}\in \left\{-1,1\right\}$ be its class membership (e.g., 1 for a normal patch and −1 for a mildew-infected patch). The objective of the SVM algorithm is to find a hyperplane determined by a normal vector w∈V and an intercept governed by b∈ℝ such that the predicted class-membership predicted by $sign\left(w^{T}f\left(x_{i}\right)+b\right)$ is correct for most data points, where $sign:\mathbb{R}\to \left\{-1,1\right\}\;$ simply denotes the sign function, and f : X →V denotes a mapping from the original feature space (i.e., the reflectance along each wave length) to a high dimensional space, implicitly defined by the kernel function as $K\left(x_{i},x_{j}\right)=f{\left(x_{i}\right)}^{T}f\left(x_{j}\right)$, where classes are presumably linearly separable. Here, the Radial Basis Kernel $K\left(x_{i},\;x_{j}\right)=exp\left(\gamma \;{\left|\left|x_{i}\;–\;x_{j}\right|\right|}^{2}\right)$ implies an implicit mapping of the feature space X to an infinite dimensional space V. The primal problem of the SVM classifier is formulated as follows:

$$\underset{w,b,z}{\min}\;\frac{1}{2}\;w^{T}w+C\;\overset{n}{\underset{i=1}{\sum }}z_{i}\;$$

$$\mathrm{subject}\;\mathrm{to}:\;\;y_{i}\left(w^{T}f\left(x_{i}\right)+b\right)\;\geq \;1-z_{i}\;\;\;\;\;\mathrm{with}\;\;\;\;z_{i}\;\geq \;\;0,\;\;\;\;\;\;\forall \;i\in \;\left\{1,\ldots ,n\right\}\;$$

This objective function aims at minimizing $w^{T}w$, the squared norm of vector w (which aims at maximizing the margin of the classifier), as well as $\overset{n}{\underset{i=1}{\sum }}z_{i}$, which is the sum of the distances between the margin boundary and the misclassified examples. Parameter C works as a regularization parameter that controls the trade-offs between the training set classification quality and the maximization of the decision function margin. High values of C tend to give more importance to the minimization of $\overset{n}{\underset{i=1}{\sum }}z_{i}$, thus allowing fewer classification errors and decreasing the margin, whereas low values of C favor the maximization of the margin, encouraging simpler models, and incurring higher misclassifications in the training set. Parameter γ controls the inverse of the radius of influence of samples selected by the model as support vectors on the classification of a data point. When γ is high, the area of influence of support vectors is small, and the model is prone to overfitting, whereas low values for γ tend to constrain the model, preventing it from learning complex shapes of classification boundaries.

To evaluate the classifier performance, we have separated, as test set instances, all the patches from one leaf per strain for every day of its infection from 1 to 9 dpi, as well as those of a control leaf for 4 days. The remaining patches were used as training set instances. We decided to separate leaves for the test set, and not separate a given number of patches possibly coming from different leaves, to prevent group data leakage and thus having a more realistic evaluation of the method [50]. Since the number of patches per leaf is not equal, our data leakage preventive test set construction lead to not exactly proportional dataset sizes, namely, the test set contained 284 patches in total (~20% of the dataset), with 106 normal patches and 178 patches infected by downy mildew, while the training set contained 1102 patches (~80% of the dataset), with 665 normal patches and 437 areas infected by downy mildew.

To tune the main hyperparameters of the SVM classifier, we relied on a grid-search cross-validation technique: we tested different combinations for the two main parameters, i.e., C and γ. In practice, we tested values for C ranging on a logarithmic grid from 1 × 10^−2^ to 1 × 10^4^, and from 1 × 10^−6^ to 1 × 10^2^ for γ. For each combination, we computed the average 10 cross-validation-weighted F1 test score. Moreover, in order to cope with the dataset-class imbalance we used a class-weighted scheme for the regularization parameter C, which was thus weighted for each class k to be inversely proportional to the class frequencies in the input data as $C_{k}=C\times n\;/\left(2\;\sum_{i=1}^{n}1_{y_{i}=k}\right)\;$, where $1_{y_{i}=k}=1$ if $y_{i}=k$ for the current class, and 0 otherwise. The parameters retained by the grid-search method are γ *= 0.005* and C *= 5000*.

## 3. Results and Discussion

### 3.1. Identification of Downy Mildew on Grapevine Leaves

In this section, firstly, we investigated the possibility of distinguishing the grapevine leaf from downy mildew. Secondly, we analysed the potential difference between several strains of *P. viticola*. If there was a difference between the strains, the detection tool would need to cope with such differences, possibly needing to detect each strain separately. However, if there was no difference, we could perform a global detection of all strains of *P. viticola*. To carry out this research, we analysed the spectral signatures of the leaves and the disease.

**Figure 3:**
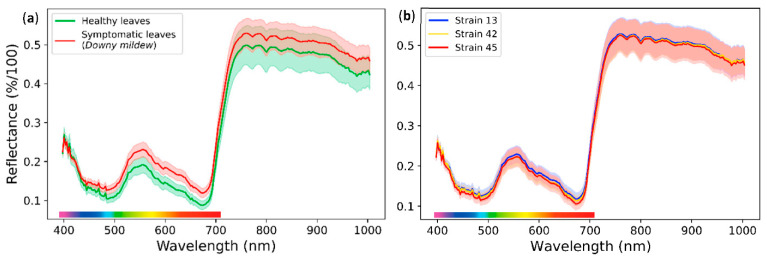
Normalized average light reflectance spectrum with standard deviation between 400 and 1000 nm of grapevine cultivar ‘Cabernet-Sauvignon’ leaf areas inoculated 9 days before with *P. viticola*. (**a**) Spectrum of healthy leaves’ areas (green) or symptomatic leaves’ areas covered by whitish downy mildew (red). Whitish downy mildew reflected more the light than a healthy leaf, especially in the visible part of the light spectrum between 400 and 700 nm; (**b**) Spectrum of symptomatic leaves’ areas covered by downy mildew strains INRA-Pv13 (blue), INRA-Pv42 (yellow) and INRA-Pv45 (red). The three strains had the same spectral signature.

The spectral data extracted from the healthy and symptomatic leaf patches provided spectral signatures of a healthy grapevine leaf and the downy mildew displayed in Figure 3a. The spectral curves have the same pattern but a different reflectance. More specifically, the downy mildew reflected more of the light than a healthy leaf, especially in the visible part of the light spectrum between 400 and 700 nm. This higher reflectance has also been observed on several grapevine cultivars inoculated with downy mildew [8,16] and on sugar beet leaves with powdery mildew [51].

Leaf optical properties are characterised by a strong absorption of light in the visible range (400–700 nm) due to photosynthetic pigments and a high reflectance in the NIR range (700–1000 nm) resulting from multiple scattering inside the intercellular air spaces of the leaf structure [10,52]. The photosynthetic pigments, particularly chlorophyll, mostly absorb blue and red light and reflect green light, which accounts for the green color of plants perceived by the human eye.

The higher reflectance of leaf tissue infected by downy mildew may be due to various causes, such as a decrease in chlorophyll content [16], but also the emergence of tufts of sporangiophores on the abaxial leaf surface [10]. Indeed, the growth of the mycelium in the leaf tissue results in the destruction of leaf cells and chlorophyll, which reduces the light absorption and increases the internal air spaces. Furthermore, the sporangiophores forming a whitish cover on the leaf surface reflect more of the light spectrum than dark colors.

The comparison between spectral signatures of the three strains INRA-Pv13, INRA-Pv42, and INRA-Pv45 showed no difference (Figure 3b). Thus, we can assume that a global detection tool for *P. viticola* could be efficient in identifying any strain of the disease. Nevertheless, it is possible to observe spectral differences between disease or plant species [8]. This must be considered for the application of such a technology in the field.

Thereby, it is possible to use their spectral signature to detect the symptoms of downy mildew on a grapevine leaf. The spectral properties for identifying *P. viticola* remain the same regardless of the strain of the disease.

### 3.2. SVM Assessment and Sensitivity Analysis

From the database of patches of healthy and infected leaves collected previously, we created an SVM classifier capable of automatically classifying patches of grapevine leaf hyperspectral images coming from healthy leaves, or from parts of leaves infected by downy mildew.

**Figure 4:**
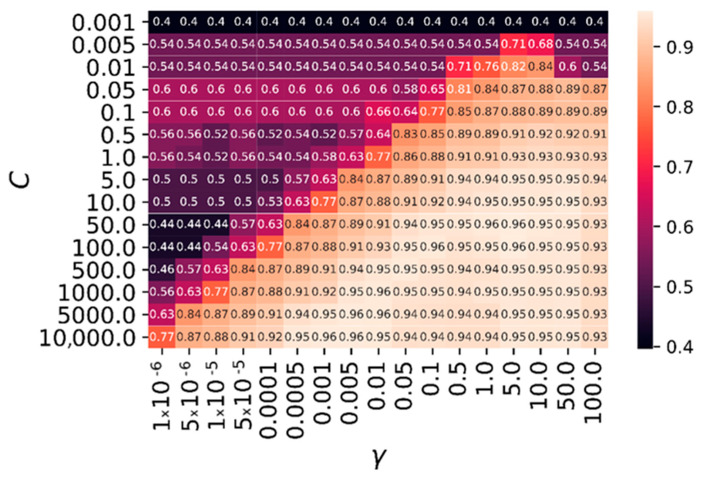
Average validation accuracy of the SVM method for a 10-fold cross-validation procedure, over different combinations of parameters *γ* (inverse kernel radius parameter) and C (regularization parameter). A large region of the parameter space allowed us to obtain good quality results with an accuracy around 0.96, meaning that the method was robust in the parameter setting.

Figure 4 depicts the average validation accuracy (similar results were observed for F1 score) of the SVM method for a 10-fold cross-validation procedure on the training dataset over different combinations of parameters *γ* and C. This figure allowed us to assess the sensitivity of the methods proposed in this paper on the main parameters, as well as to justify the parameter setting.

In general, the performance of the algorithm tended to increase when *γ* and C increased, since the model was more flexible and less prone to underfitting. Nevertheless, when *γ* was too high (here, when *γ = 100*), the performance of the model in the validation set dropped, which was likely to be due to overfitting. Similarly, when parameter C was too high, it could slightly reduce the performance of the SVM classifier. A rather large region of the parameter space allowed us to obtain good quality results with an accuracy around 0.96 (i.e., on average, 96% of the validation patches were correctly classified), meaning that the method was robust in the parameter setting.

To assess the performance of the algorithm on unseen data, we applied the SVM method on the test set, and we evaluated the results by computing the corresponding confusion matrix depicted in Table 2. The results depicted in this table show that most test patches were correctly classified by the SVM. Only one infected leaf region patch, and two healthy leaf patches were wrongly classified, while all the other 321 patches were correctly classified. The SVM obtained high quality results on the test set with an accuracy score of 0.99 (i.e., 99% of the test patches were correctly classified), meaning that the method was also efficient with unseen data.

By comparison, a few studies using HSI and classification models based on discriminant analysis to detect infected grapevine bunches reached an accuracy of 99% and 85% in the cross-validation model, respectively [38,39], and an accuracy for the test set of 87% for entire-bunch classification [38] and around 76% for pixel classification [39]. Other studies using SVM [53] and Spectral Angle Mapper (SAM) [29] classifiers to detect powdery mildew on sugar beet leaves achieved 93% and 90–97% accuracy depending on the stage of disease development, respectively.

### 3.3. Early Detection of the Disease

**Figure 5:**
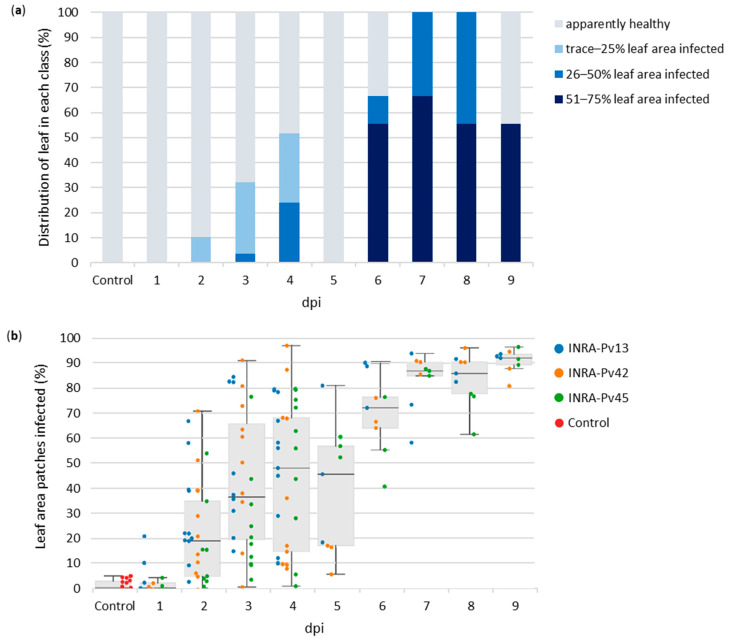
Visual assessment (**a**) and automatic detection (**b**) of areas infected by the three strains of downy mildew on grapevine leaves. (**a**) Leaves were classified every day by disease severity scale as a percentage of leaf area visually infected by downy mildew; (**b**) Infected leaf area rate over time automatically detected by the SVM classifier. Each colored point corresponds to the area of one leaf infected by a downy mildew strain. The percentage of infected area was calculated by randomly selecting 5000 patches on the leaf, keeping the ones that only contain leaf pixels, computing their average spectrum and then classifying them as healthy or infected. Thus, for instance the patches containing the edges of the leaf and part of the background were removed and were not considered in the percentage of infected area approximation.

To further evaluate the quality of this automated detection system, we compared expert visual assessment of downy mildew on grapevine leaves, classified in four disease severity scales as a percentage of leaf area infected (Figure 5a), with the infected leaf area rate detected by the SVM classifier (Figure 5b). This comparison was carried out every day from 1 to 9 dpi, thus enabling highlighting of a possible early detection of the disease by the SVM classifier.

First, the SVM classifier detected 0–5% of downy mildew-infected area on control leaves, which could be considered as healthy leaves (Figure 5b). Indeed, as previously proposed on wine bunches in the literature [39], it is possible to establish a tolerance threshold denoting that leaves with less than 10% of leaf area classified as infected can be considered healthy.

Then, according to Figure 5a, the visual assessment allowed clear detection of downy mildew on a few grapevine leaves only from 4 dpi, corresponding to 26–50% of leaf area infected. Before that, we only saw traces of the disease on some leaves at 2 and 3 dpi. In comparison, the SVM classifier detected downy mildew on most leaves from 2 dpi with a median infected leaf area of 20%, then close to 40% at 3 dpi and 50% at 4 dpi.

Due to the brightness of the RGB images transmitted by the camera being too low on day 5 to see the downy mildew, we noted no infection at 5 dpi. However, the SVM classifier successfully detected downy mildew with a median infected leaf area around 50%, thanks to the spectral information of the normalized image.

From 6 to 9 dpi, most of the leaves were visually assessed as highly infected with 51–75% leaf area infected, with the other leaves being at 26–50% leaf area infected, or not apparently infected for a few samples proving the visual assessment to be challenging [38]. The SVM classifier detected a high disease severity on almost all leaves with a median infected leaf area of 70% at 6 dpi and around 90% after.

Thus, the SVM classifier could automatically detect downy mildew on most grapevine leaves at 2 dpi, 2 days before the clear appearance of the visual symptoms on a few leaves at 4 dpi. Moreover, thanks to spectral analysis, it could detect larger areas of downy mildew on leaves than visual assessment and was more accurate about the actual disease severity on the grapevine leaves. However, the SVM classifier detection excluded the patches of the edges of the leaf, containing a part of background, which were not considered in the percentage of infected area approximation, which slightly reduced the leaf area analysed.

Other studies using fluorescence [6] and thermal imaging [14] to early detect downy mildew on grapevine leaves of Chardonnay and Riesling cultivars, whose susceptibility to disease is similar to ‘Cabernet-Sauvignon’ [3,54], have detected the first symptoms of downy mildew only at 3–4 dpi.

### 3.4. Assessment of Disease Severity over Time via Spatial Distribution of Downy Mildew on Leaves

**Figure 6:**
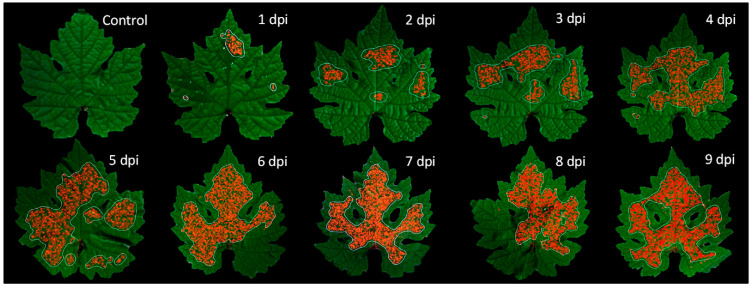
Spatial distribution of downy mildew strain INRA-Pv45 over a grapevine leaf from 1 to 9 dpi, automatically detected by the SVM classifier. The leaf remained unknown to the classifier, as a test set. Positive patches of downy mildew are represented by red crosses. The downy mildew was slightly detected at 1 dpi, then three zones of infection were clearly detected at 2 dpi, corresponding to the three drops of inoculum deposited on the leaf on day 0. The infected area grew over time and covered almost the entire leaf at 9 dpi, except leaf borders which were not considered in the detection.

The analysis of many leaf area patches by the SVM classifier made it possible to show the spatial distribution of downy mildew on leaves. In Figure 6, one example of an infected leaf from the test set (which remained unknown to the classifier) is shown from 1 to 9 dpi next to a control leaf from the test set. All the patches classified as downy mildew were marked with red crosses on RGB images of the leaf.

The spatial distribution of downy mildew over time in Figure 6 is coherent with the global rate of infected leaf area detected by the SVM classifier in Figure 5b. Indeed, as in Figure 5b, the spatial distribution showed no downy mildew for the control leaf and just slight spots at 1 dpi. Then, at 2 dpi, three areas of downy mildew could be clearly identified, which corresponded to the three drops of inoculum deposited on the leaf initially. Thus, as shown in Figure 5b, the SVM classifier could reveal in Figure 6 an early spatial distribution of downy mildew on grapevine leaves 2 days before the onset of visual symptoms at 4 dpi (Figure 5a). Afterwards, the infected area grew over time and covered almost the entire leaf at 9 dpi.

In comparison with the literature, the spatial representation of powdery mildew on vine bunches [38,39] and sugar beet leaves [29] based on classification results had been made with great results despite some false positives in healthy samples due to peripheral zones, likely producing disparate spectral signatures [39]. This confirms our choice not to consider leaf borders in our analysis.

## 4. Conclusions

First, by analysing their spectral signature extracted from hyperspectral images, we observed that the three strains of downy mildew considered here had the same spectral signature and we clearly differentiated downy mildew than healthy grapevine leaves. Then, we built an SVM classifier based on a spatial–spectral database of infected and healthy patches, trained it using a 10-fold cross-validation technique, and obtained promising validation average accuracy scores of 0.96, and a test accuracy score of 0.99.

The SVM classifier automatically detected the presence and the disease severity of downy mildew on grapevine leaves over time. The disease severity was assessed as a percentage of leaf area infected and was illustrated in parallel by spatial distribution over the leaves. The SVM classifier showed promising results in comparison to the visual assessment of downy mildew disease severity scales on leaves. Considering a minimum threshold of 10% leaf area infected, the SVM classifier did not output false positives on the control leaves. Then, it detected downy mildew on most leaves at 2 dpi, 2 days before the clear onset of visual symptoms on a few leaves at 4 dpi, and the disease area detected over time was higher than when visually assessed, providing a more accurate evaluation of disease severity.

To our knowledge, this is the first study using HSI to automatically detect and show the spatial distribution of downy mildew on grapevine leaves early over time. Thus, automated detection tools based on hyperspectral image analysis can be a non-destructive and reproducible technique to detect diseases in grapevines early with a good level of accuracy. Additional research would be required to test the application of such innovative tools in the field.

## Figures and Tables

**Table 1:** Disease severity scale as a percentage of leaf area infected, adapted from [9].

Category	Severity
0	apparently healthy
1	trace-25% leaf area infected by downy mildew
2	26–50% leaf area infected by downy mildew
3	51–75% leaf area infected by downy mildew

**Table 2:** Confusion matrix corresponding to the SVM method results on the test set. Only one patch corresponding to an infected leaf region was classified as healthy, while two patches corresponding to healthy leaves were classified as patches corresponding to infected regions. All other patches were correctly classified.

		Predicted Label	
		Infected (Downy mildew)	Healthy
**True label**	Infected (Downy mildew)	177	1
	Healthy	2	144

## Data Availability

The hyperspectral images used in this work came from a database publicly available [40] at https://doi.org/10.57745/AV1ETI, accessed on 19 July 2022.
